# Novel targets in renal fibrosis based on bioinformatic analysis

**DOI:** 10.3389/fgene.2022.1046854

**Published:** 2022-11-29

**Authors:** Yuan Yuan, Xi Xiong, Lili Li, Pengcheng Luo

**Affiliations:** ^1^ Department of Urology, Wuhan Third Hospital and Tongren Hospital of Wuhan University, Wuhan, China; ^2^ Department of Urology, Wuhan Third Hospital School of Medicine, Wuhan University of Science and Technology, Wuhan, China; ^3^ Central Laboratory, Renmin Hospital of Wuhan University, Wuhan, China

**Keywords:** chronic kidney disease (CKD), renal fibrosis, protein-protein interaction (PPI), hub genes, bioinformatics analysis

## Abstract

**Background:** Renal fibrosis is a widely used pathological indicator of progressive chronic kidney disease (CKD), and renal fibrosis mediates most progressive renal diseases as a final pathway. Nevertheless, the key genes related to the host response are still unclear. In this study, the potential gene network, signaling pathways, and key genes under unilateral ureteral obstruction (UUO) model in mouse kidneys were investigated by integrating two transcriptional data profiles.

**Methods:** The mice were exposed to UUO surgery in two independent experiments. After 7 days, two datasets were sequenced from mice kidney tissues, respectively, and the transcriptome data were analyzed to identify the differentially expressed genes (DEGs). Then, Gene Ontology (GO) and Kyoto Encyclopedia of Genes and Genomes (KEGG) analysis were executed. A Protein-Protein Interaction (PPI) network was constructed based on an online database STRING. Additionally, hub genes were identified and shown, and their expression levels were investigated in a public dataset and confirmed by quantitative real time-PCR (qRT-PCR) *in vivo.*

**Results:** A total of 537 DEGs were shared by the two datasets. GO and the KEGG analysis showed that DEGs were typically enriched in seven pathways. Specifically, five hub genes (Bmp1, CD74, Fcer1g, Icam1, H2-Eb1) were identified by performing the 12 scoring methods in cytoHubba, and the receiver operating characteristic (ROC) curve indicated that the hub genes could be served as biomarkers.

**Conclusion:** A gene network reflecting the transcriptome signature in CKD was established. The five hub genes identified in this study are potentially useful for the treatment and/or diagnosis CKD as biomarkers.

## Introduction

In recent decades, CKD has been a severe threat to human health globally (Hill et al., 2016). Indeed, over 10% of adults living in developed countries are exposed to CKD (Global, 2015; Bach et al., 2019). An indicator of CKD is the presence of fibrosis and renal failure induced by fibrosis.

As a typical histopathological characteristic of chronic obstructive nephropathy, renal fibrosis serves as a terminal pathway of most progressive and chronic nephropathies. As is known, fibrosis is essentially pathologically related to the wound healing process accompanied by migration and activation of myofibroblasts (Humphreys, 2018). Despite great advances in the molecular mechanisms of CKD progression over the past years, few effective therapeutic strategies have been applied clinically. Therefore, a thorough investigation of renal fibrosis related molecular mechanisms is urgently required (Djudjaj and Boor, 2019). Additionally, the molecular basis of CKD provides references to the enhancement of therapeutic intervention and clinical diagnosis.

Omics approaches, including metabolomics, proteomics, transcriptomics and genomics, serve as high-throughput methods to investigate the changes of global gene expressions in CKD patients (Eddy et al., 2020). Next-generation sequencing-based transcriptome profiling is a commonly applied method for identification of DEGs. To date, various functional pathways and DEGs have been identified by means of bioinformatic analysis. A previous study analyzed by bioinformatics and identified 44 genes possibly related to CKD (such as ACTN4) that affect CKD through SMAD2/3 signal transduction pathway (Yu et al., 2020). Another study also identified four CKD related genes, PLG, ITGB2, CTSS, and CCL5, through bioinformatics analysis. These studies improve the understanding of the transcriptome status of CKD and provides directions for the further investigations of the mechanisms of CKD (Wang et al., 2020). Nevertheless, no reliable biomarker for CKD diagnosis has been reported. Bioinformatic analysis integrating such data of expression profiling may be an effective measure.

To address this issue, we sequenced and analyzed two transcriptional datasets from mouse kidney tissue samples under UUO surgery. A total of 537 DEGs shared by the two datasets were identified. Meanwhile, GO, KEGG pathway enrichment, and PPI network were achieved to clarify the role of DEGs in CKD. Additionally, hub genes were identified and confirmed. This study provides facilitates identification of the molecular targets for CKD treatment.

## Materials and methods

### Animal model

Mice (C57BL/6N, male) aged 8 weeks were supplied by Vital River Laboratory Animal Technology Co., Ltd. (China) and kept in the Central Laboratory of Wuhan Third Hospital (China). In the first study, ten mice were equally divided into the sham group and the UUO group on a random basis. In the second study, eight mice were equally divided into the sham group and the UUO group on a random basis. The experiments were reviewed and approved by the authority (^#^SY2021-020) and executed in accordance with relevant guidelines.

### Unilateral ureteral obstruction surgery

The mice were exposed to right ureter ligation before being divided into the sham operated group and the 7 days ligated group. Specifically, the mice were anesthetized by using a face mask that delivers 30% isoflurane. The exposure of the right ureter was achieved by using a midline abdominal incision. After that, the right ureter was completely obstructed (1 cm below the renal pelvis with 5.0 silk ligature, in the 7 days ligated group) or manipulated in a similar way instead of being ligated (the sham operated group). Seven days later, the mouse kidneys were collected, followed by rinsing with isotonic saline. Finally, the mouse kidneys were dissected and kept in liquid nitrogen.

### Immunofluorescence

Frozen sections of kidneys were cut at the 5-µm thickness section for subsequent proteins staining of extracellular matrix (ECM). These sections were immersed in a protein-blocking solution, followed by incubation with primary collagen I antibody (Servicebio, ^#^GB11022-3, Wuhan, China), fibronectin antibody (Servicebio, ^#^GB13091, Wuhan, China), and E-cadherin antibody (Sevicebio, ^#^GB13083, Wuhan, China). Afterwards, the sections were sequentially incubated with appropriate secondary antibodies. Additionally, 25 µl DAPI was dropped on the samples and kept for 5 min. Utilizing a fluorescence microscope with a digital camera, image visualization was achieved. Using ImageJ according to the formula below: mean of the average fluorescence intensity = total fluorescence intensity of this region (INTDEN)/area, the average fluorescence intensity was determined.

### Transcriptional sequencing

The transcriptional sequencing data were sequenced in Beijing Genomic institute (BGI, Shenzhen, China). Total RNA extraction was achieved by utilizing Trizol, followed by qualification and quantification analysis by utilizing a Nano Drop and bioanalyzer (Agilent 2100, Thermo Fisher Scientific). Additionally, the data were sequenced by using the MGISEQ2000 platform. In Study 1, dataset 1 containing 5 sham and 5 UUO samples was generated. In Study 2, dataset 2 containing 4 sham and 4 UUO samples was generated.

### Identification of differentially expressed genes

The DEGs shared by the two groups were analyzed in the two datasets by using the Limma package in R. The critical level for the DEGs selection with statistical significance was set as |log2FC (fold change) | > 2 and *p* < 0.05. The shared DEGs were identified by drawing the Venn diagram.

### Kyoto encyclopedia of genes and genomes and gene ontology enrichment analyses of differentially expressed genes

For the functions of DEGs, KEGG pathway enrichment and GO analysis [e.g., molecular function (MF), cellular components (CC) and biological process (BP)] were executed using the R package clusterProfiler.

### Establishment of the protein-protein interaction network

In this study, prediction of the PPI network was achieved on the basis of an online database called the Search Tool for the Retrieval of Interacting Genes (STRING) (http://string-db.org) (Szklarczyk et al., 2017). The critical value for STRING was 0.9. Investigations of functional PPI facilitate understanding of the biological mechanisms. Additionally, the PPI network was visualized and investigated by utilizing Cytoscape (version 3.4.0) (Chin et al., 2014), which has been employed for the clustering of a specific network on the basis of topology; the dominant modules were also identified in the PPI network. Five hub genes were identified by performing the 12 scoring methods in cytoHubba.

### Analysis of hub genes

The PPI network of the identified hub genes were investigated by utilizing the database STRING. The expression levels of these hub genes were confirmed in the dataset GSE36496, which is available in the GEO database.

### RNA extraction and quantitative real-time PCR

The total mRNA of the renal tissue samples was extracted by RNA isolation kit (Omega Bio-Tek, #R6934-01, United States), and 1 μg of mRNA was reversely transcribed into cDNA. The mRNA expression levels of target genes were detected by qRT-PCR. Molecular markers of renal fibrosis, including Fibronectin, E-cadherin, and collagen I, were detected with β-actin as an internal reference gene. Table 1 lists the primer sequences.

**TABLE 1:** The primer sequences of 5 hub genes.

Gene	Forward primer (5'-3')	Reverse primer (5'-3')
Bmp1	GAA​CAC​ATT​CTC​CAG​GGG​CA	CTT​GCT​GAG​TCG​GGT​CCT​TT
CD74	GGA​TGG​CGT​GAA​CTG​GAA​GA	CCT​GGC​ACT​TGG​TCA​GTA​CTT​T
Fcer1g	CAG​CCG​TGA​TCT​TGT​TCT​TGC	CCT​TTC​GGA​CCT​GGA​TCT​TGA
Icam1	AGC​CTC​CGG​ACT​TTC​GAT​CT	TGT​TTG​TGC​TCT​CCT​GGG​TC
H2-Eb1	ACG​GTG​TGC​AGA​CAC​AAC​TA	GTC​ACC​GTA​GGC​TCA​ACT​CT

### Receiver operating characteristic curve

The diagnostic value of the five hub genes for CKD was evaluated over the two datasets by using the R package pROC and the results were indicated by the receiver operating characteristic (ROC) curves.

## Results

### Immunofluorescence

**FIGURE 1:**
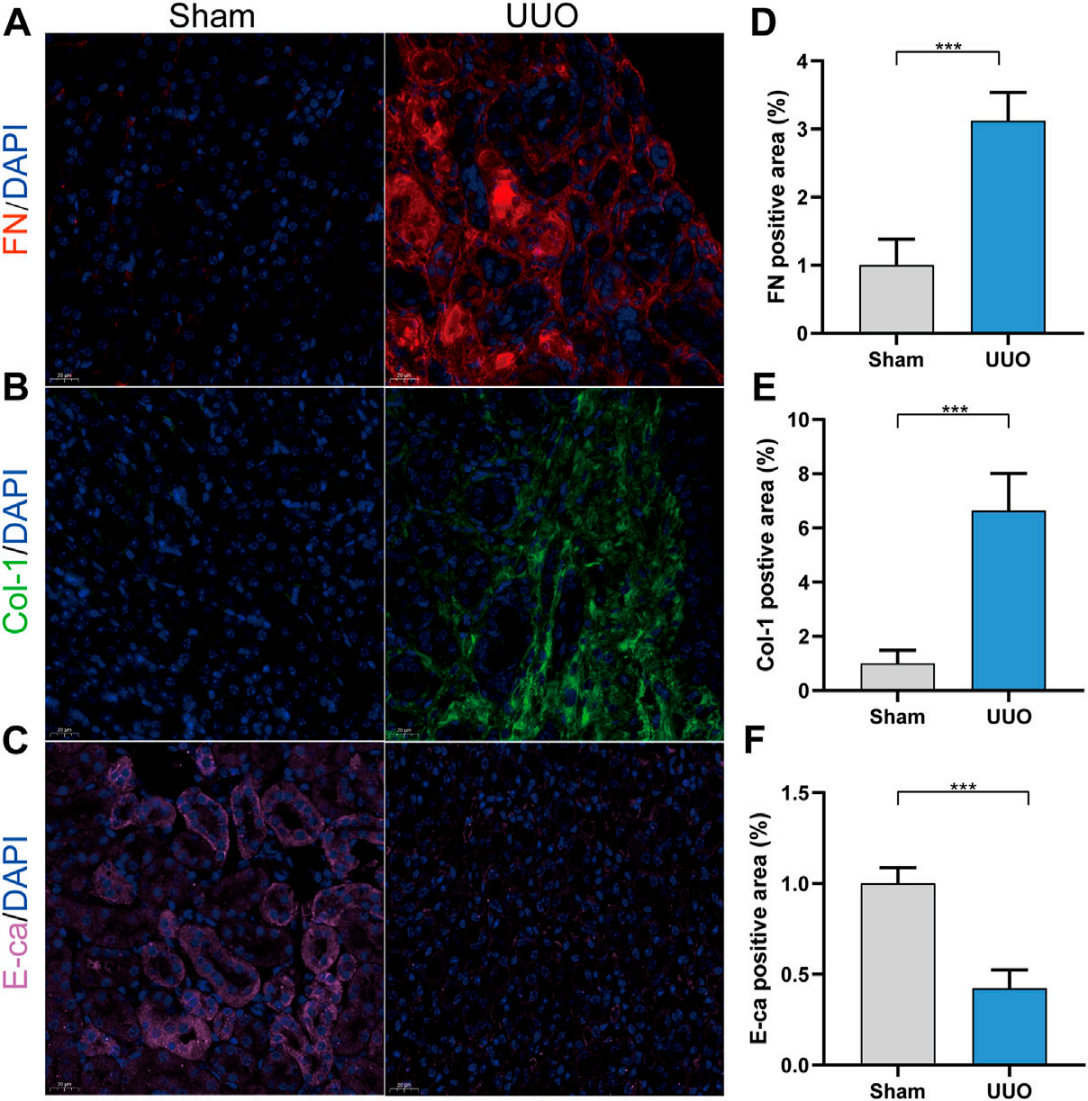
UUO model was conducted successfully. **(A)** Representative immunofluorescence of kidney sections stained with Fibronectin (red) and DAPI (blue). **(B)** Representative immunofluorescence of kidney sections stained with collagen I (green) and DAPI (blue). **(C)** Representative immunofluorescence of kidney sections stained with E-cadherin (pink) and DAPI (blue). **(D)** Quantitative analysis of the fibronectin positive area in the kidney samples. **(E)** Quantitative analysis of the collagen I positive area in the kidney samples. **(F)** Quantitative analysis of the E-cadherin positive area in the kidney samples. ***p < 0.001.

The expressions of fibronectin and collagen I were significantly up-regulated in the UUO group, while E-cadherin expression was significantly down-regulated, compared with the sham group (Figure 1). According to the immunofluorescence results, we could observe that renal fibrosis was aggregated in the UUO model.

### Identification of differentially expressed genes

**FIGURE 2:**
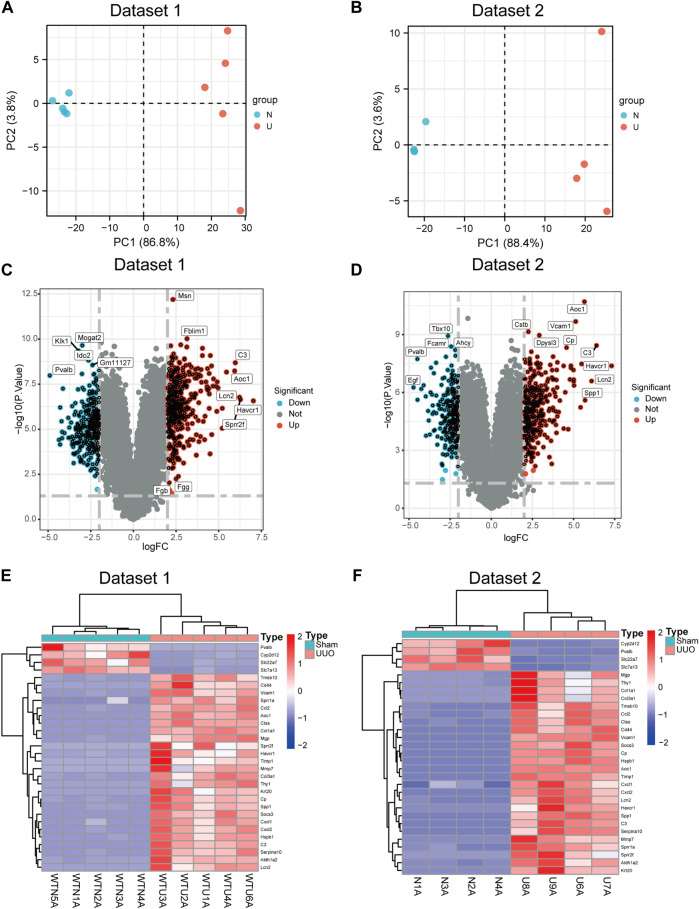
DEGs were identified between sham and UUO. **(A)** Principal component analysis (PCA) plot of dataset 1. **(B)** PCA plot of dataset 2. **(C)** Volcano plot of DEGs in dataset 1. **(D)** Volcano plot of DEGs in dataset 2. **(E,F)** Heatmap showing the gene expression of top 30 DEGs in dataset 1 **(E)** and in dataset 2 **(F)** Each row represents a gene and each column represents a sample.

The two transcriptome datasets were obtained by high throughput sequencing. The two groups exhibited distinctive expressions, as indicated by the PCA plots of the two datasets (Figures 2A,B). Meanwhile, volcano plots of DEGs were generated in both datasets (Figures 2C,D) based on the criteria of |Log2FC| > 2 and *p* < 0.05. The expression level of top 30 DEGs in datasets 1 and 2 were shown in Figures 2E,F.

### Gene ontology and kyoto encyclopedia of genes and genomes enrichment analyses of the shared differentially expressed genes

**FIGURE 3:**
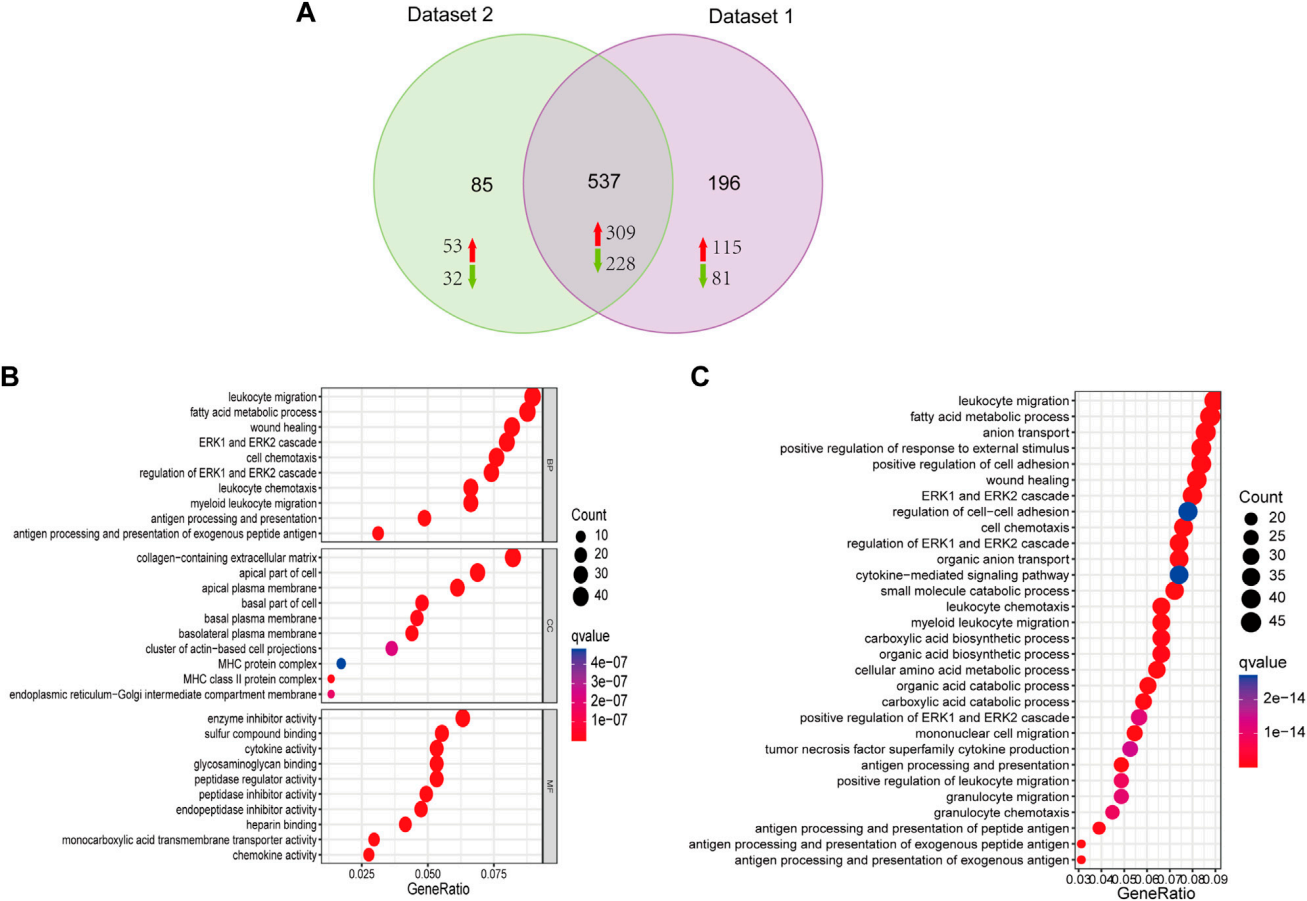
Functional analysis of DEGs. **(A)** Venn diagram showing the 537 shared DEGs from the two datasets, including 309 upregulated and 228 downregulated genes. **(B)** Biological process (BP), cellular components (CC), and molecular function (MF) are the components of GO enrichment analysis results, and each part displays the top ten GO terms. **(C)** KEGG pathway enrichment of DGEs.

According to the Venn diagram, 537 shared DEGs were identified in two datasets, including 309 upregulated and 228 downregulated genes. In dataset 1, 733 significant DEGs were identified, including 424 upregulated and 309 downregulated genes. In dataset 2, 622 significant DEGs were identified, including 362 upregulated and 260 downregulated genes (Figure 3A). GO and KEGG analyses were employed to clarify the biological function of shared DEGs. According to the GO results, we observed that BP enriched in leukocyte migration, fatty acid metabolic process, wound healing and ERK1 and ERK2 cascade; CC enriched in apical plasma membrane, ECM containing collagen, and apical part of cells; MF enriched in enzyme inhibitor activity, sulfur compound blinding, cytokine activity, glycosaminoglycan binding and peptidase regulator activity (Figure 3B). According to the KEGG result, the enrichment of DEGs was dominantly observed in leukocyte migration, fatty acid metabolic process, anion transport and positive regulation of response to external stimuli (Figure 3C).

### Protein-protein interaction network and hub genes

**FIGURE 4:**
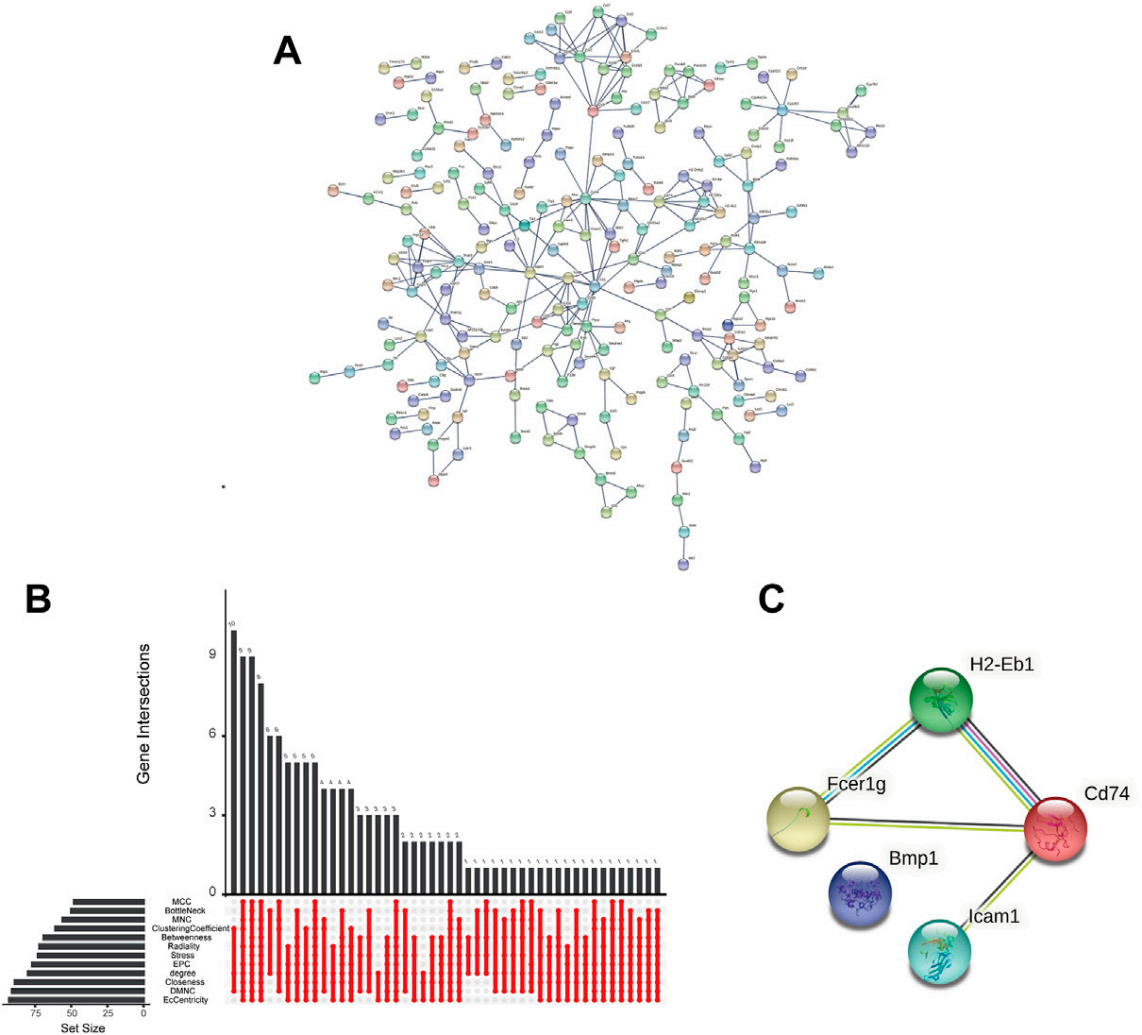
Interaction network and biological process analysis of the hub genes. **(A)** PPI network of shared DEGs constructed by STRING. **(B)** The performance of 12 scoring methods in selecting 5 hub genes by cytoHubba. **(C)** The PPI network of hub genes constructed by STRING.

A PPI network was constructed for the 537 shared DEGs by utilizing the database STRING (Figure 4A). Meanwhile, cytoHubba was employed to investigate the network comprised of the hub genes and co-expression genes. Five hub genes (Bmp1, CD74, Fcer1g, Icam1, and H2-Eb1) were identified by performing the 12 scoring methods in cytoHubba, as well as analysis by UpSetR (Figure 4B). The PPI of five genes were shown in Figure 4C. Table 2 lists the information (e.g., names, abbreviations and functions) of these genes. The established PPI network indicated the functional connection among genes (Figure 4C). Function information acquired from NCBI suggested that the hub genes are proinflammatory and profibrosis related genes.

**TABLE 2:** The functions of five hub genes.

Gene	Full name	Function^a^
Bmp1	Bone Morphogenetic Protein 1	Metalloprotease that plays key roles in regulating the formation of the extracellular matrix (ECM) *via* processing of various precursor proteins into mature functional enzymes or structural proteins
CD74	CD74 Molecule	Plays a critical role in MHC class II antigen processing by stabilizing peptide-free class II alpha/beta heterodimers in a complex soon after their synthesis and directing transport of the complex from the endoplasmic reticulum to the endosomal/lysosomal system where the antigen processing and binding of antigenic peptides to MHC class II takes place
Fcer1g	Fc Epsilon Receptor 1g	Adapter protein containing an immunoreceptor tyrosine-based activation motif (ITAM) that transduces activation signals from various immunoreceptors
Icam1	Intercellular Adhesion Molecule 1	ICAM proteins are ligands for the leukocyte adhesion protein LFA-1 (integrin alpha-L/beta-2). During leukocyte trans-endothelial migration, Icam1 engagement promotes the assembly of endothelial apical cups through ARHGEF26/SGEF and RHOG activation
H2-Eb1	orthologous gene of human HLA-DRB1	The human major histocompatibility complex (MHC), which is also called human leucocyte antigen (HLA), of which HLA-DRB1 is one of the functional MHC-II genes that is involved in encoding the chain of the HLA-DR structure in the antigen binding slot

^a^ Information was from NCBI (https://www.ncbi.nlm.nih.gov/).

### Expressions of hub genes in the two datasets

**FIGURE 5:**
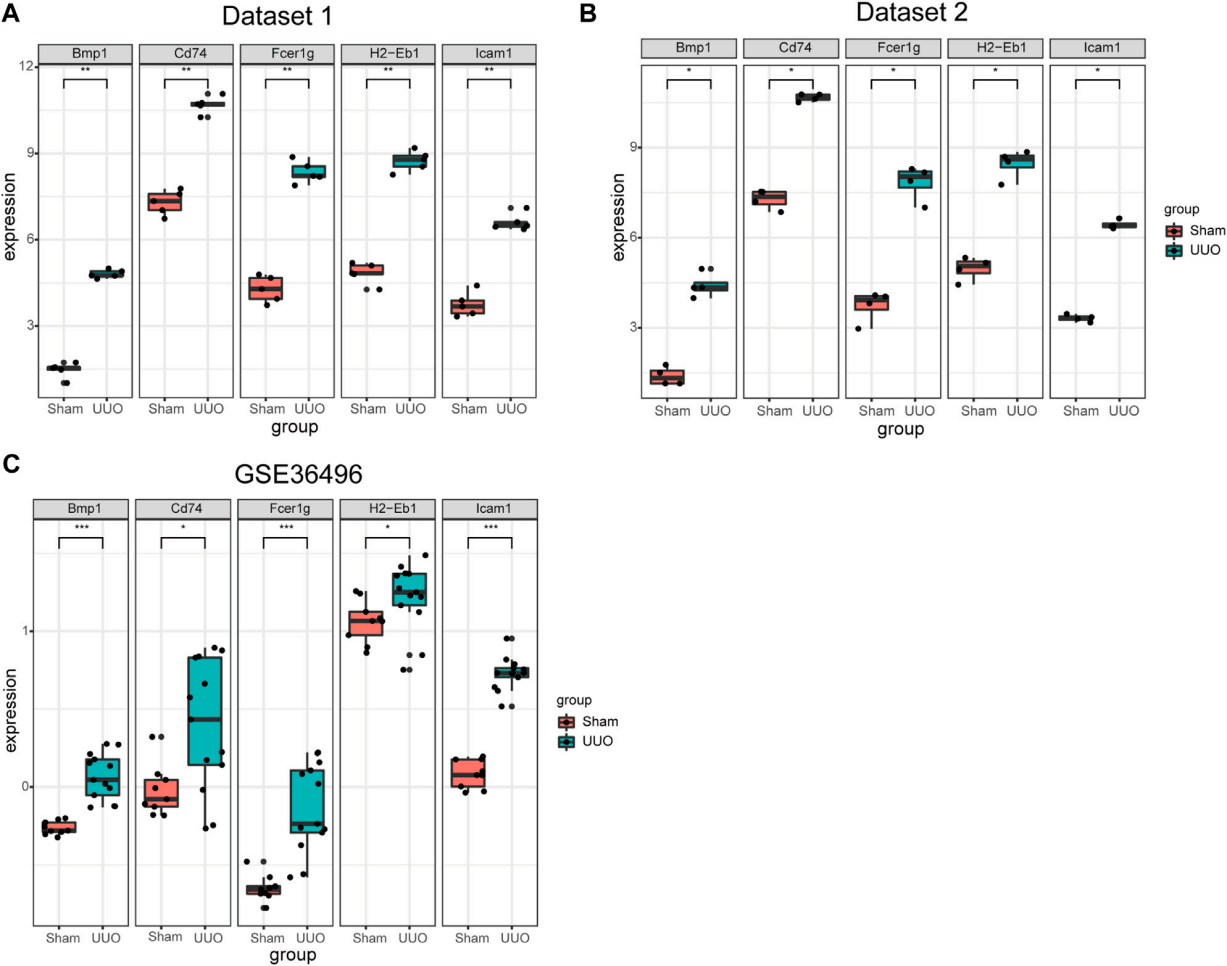
The expression of the five hub genes in three datasets. **(A)** The expression of the five hub genes in dataset 1. **(B)** The expression of the five hub genes in dataset 2. **(C)** The mRNA expression level of the hub genes in dataset GSE36496. *p < 0.05, **p < 0.01, ***p < 0.001.

**FIGURE 6:**
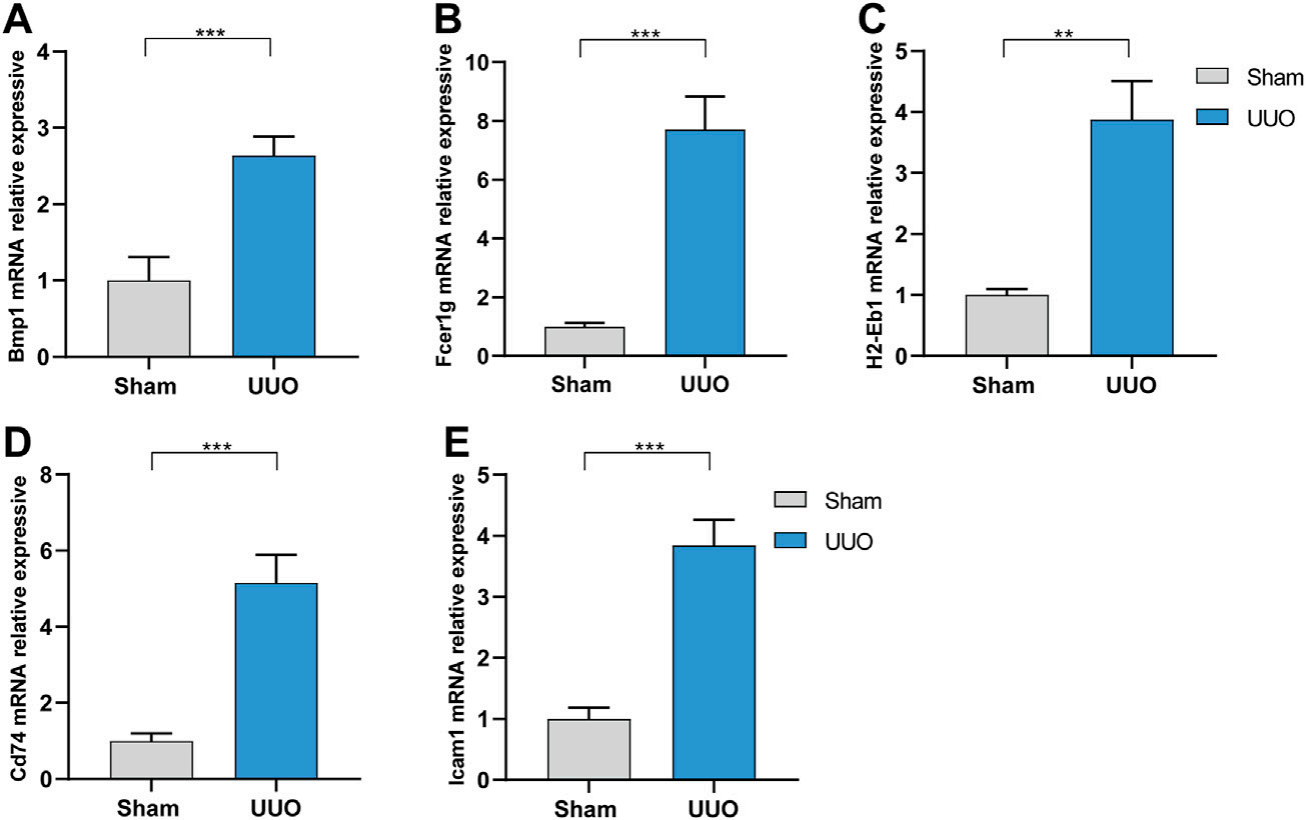
The mRNA expression level of the hub genes *in vivo* analyzed by qRT-PCR in dataset 1. **(A)** Bmp1. **(B)** Fcer1g. **(C)** H2-Eb1. **(D)** CD74. **(E)** Icam1. **p < 0.01, ***p < 0.001.

Expressions of the five hub genes in the UUO group were significantly up-regulated in both datasets compared with the sham group (Figures 5A,B). Meanwhile, the expression of the five hub genes in dataset GSE36496 was validated. The results showed that the expressions of the five genes were significantly up-regulated after the UUO surgery (Figure 5C). To confirm the mRNA expression levels *in vivo*, we performed qRT-PCR to detected the mRNA expression of hub genes in kidney samples collected from the dataset 1 (Figure 6). As indicated, the expressions of the five hub genes in UUO kidney tissues were significantly up-regulated compared with those in sham kidney tissues.

**FIGURE 7:**
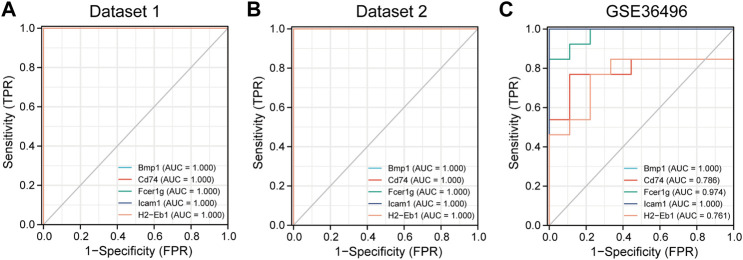
The receiver operating characteristic (ROC) curve of the five hub genes in three datasets. **(A)** The diagnosis value of the 5 hub genes in dataset 1. **(B)** The diagnosis value of the 5 hub genes in dataset 2. **(C)** The diagnosis value of the 5 hub genes in dataset GSE36496. AUC: area under the curve.

The diagnostic value of the five hub genes for CKD was evaluated. The ROC curves showed that the area under the curve (AUC) of the five hub genes for the the three datases was ranged 0.761 to 1.000 (Figure 7), suggesting that the value of these hub genes could be used for the diagnosis of CKD. In summary, the five hub genes are potential biomarkers in CKD.

## Discussion

As one of the dominant outcomes of CKD, renal fibrosis affects approximately 10% of the global population and has a direction correlation with the annually increasing deaths and healthcare cost (Glassock et al., 2017). At the early stage, renal fibrosis seems to be no more than a normal response to injury. Herein, excess ECM was generated by activated fibroblasts during a wound-healing process to facilitate tissue repair. For repetitive injury, ECM accumulation induced by chronic wound healing was observed, resulting in organ dysfunction (Gurtner et al., 2008). Indeed, renal fibrosis is of great significance to the CKD in terms of pathology and its progression to the ESRD. Over the past years, great efforts have been devoted to identification of novel effective mediators and targets for CKD therapy, while current therapeutic interventions are insufficient. Therefore, novel gene targets could be identified by using high-throughput sequencing.

In this study, two datasets were employed to analyze hub genes correlated with renal fibrosis. A total of 537 shared DEGs were identified (Figure 4A). Five hub genes (Bmp1, CD74, Fcer1g, ICAM-1 and H2-Eb1) were identified (Figures 4C,D). As the five hub genes are excessively expressed in UUO tissues, the characteristics of hub genes are significantly related to the renal fibrosis process. Function information from NCBI indicated that the hub genes belong to proinflammatory genes and profibrosis genes. GSE36496 dataset validation (Figure 5C), qRT-PCR confirmation results (Figure 6), and the ROC curves (Figure 7) suggested that the five hub genes are potential biomarkers for CKD.

Bmp1, which is also denoted as the procollagen C-propeptide protease (Tabas et al., 1991; Kessler et al., 1996) is a member of the peptidase M12A family of BMPs triggering development of bone and cartilage. Different from other BMPs, Bmp1 is not a member of the TGF-β superfamily (Panickar et al., 2020). It exhibits features of metal protease (matrix metalloproteinases), which can cut the COOH-terminals of Procollagen I, II and III, resulting in collagen maturation and ECM deposition (Hopkins et al., 2007; Rosell-Garcia and Rodriguez-Pascual, 2018). Grgurevic *et al.* claimed that Bmp1 has a positive effect on the cleavage of procollagen I, resulting in deposition of collagen in cirrhosis (Grgurevic et al., 2017). Additionally, Bmp1-3 can accelerate renal fibrosis in the mouse model of CKD by increasing the deposition of ECM attenuated by polyclonal antibodies specifically against Bmp1 or Bmp3 (Grgurevic et al., 2011). As is known, Bmp1 is a fundamental part of the development and formation of ECM (Bai et al., 2019; Correns et al., 2021). In summary, Bmp1 may serve as a target for renal fibrosis therapy.

CD74 (MHCII class invariant chain, II) is a nonpolymorphic transmembrane glycoprotein II. In addition to its regulatory role in MHCII expression on the cell surface, CD74 exhibits various biological functions under different pathological and physiological conditions and is involved in the transport of other non-MHCII proteins (Su et al., 2017). Meanwhile, CD74 molecule is a cell membrane receptor with high affinity to bacterial protein, d-dobutamine tautomeric isomerase (D-DT/mif-2) and macrophage migration inhibitory factor (Bilsborrow et al., 2019). CD74 is also an auxiliary signal molecule with regulated intramembrane proteolysis upon ligand binding (Kupke et al., 2020). Additionally, CD74 is critical in various inflammatory diseases (Valiño-Rivas et al., 2015), including Alzheimer disease, systemic lupus erythematosus, type I diabetes and liver fibrosis (Su et al., 2017). Indeed, CD74 generates a proinflammatory response in renal cells under activation by macrophage migration inhibitory factor (MIF) (Farr et al., 2020). For this reason, CD74 may modulate homeostasis and tissue injury besides its impacts on immune regulation. In this study, CD74 was closely correlated with the process of renal fibrosis and could be a biomarker for mechanism studies in the future.

Located in the chromosome 1q23.3, fcer1g, also known as FcRγ, encodes the cytoplasmic FcRg of immunoglobulin. Meanwhile, Fcer1g is constitutively generated by macrophages and monocytes. When stimulated with IL-12, IFN-α, granulocyte colony-stimulating factor, and IFN-γ (Bournazos et al., 2016), the expression of Fcer1g may be induced in other myeloid cells. Also, this is related to the proinflammatory phenotype of macrophages. Fcer1g is essentially a signal-transducing subunit with an immunoreceptor tyrosine-based activation motif (ITAM) and it is responsible for the transduction of activation signals acquired from immune receptors (Shah et al., 2017), which play key roles in different pathological processes, including chronic inflammatory programs, immune cell activation, and allergic reactions (Andreu et al., 2010). As indicated, Fcer1g is related to various kidney diseases, e.g., clear cell renal cell carcinoma and diabetic kidney disease (Chen et al., 2017; Liu et al., 2020). Recently, Fcer1g has been identified to be a biomarker of inflammatory dendritic cells as it is excessively expressed in the periglomerular region of the lupus nephritis kidney (Parikh et al., 2021). Combined with the findings of the current research, it is suggested that Fcer1g has a close relationship with the progress of renal fibrosis and is a potential target for CKD treatment.

Icam1 (Inter cellular adhesion molecule-1) is an adhesion receptor found in leukocytes and endothelial cells. Previous reports have shown that upregulation of both Icam1 and Vcam1 had a positive correlation with the destruction of capillaries and tubular cells in kidney rejection (Vitoria et al., 2019; Zhang et al., 2021). On one hand, adhesion molecules serve as infiltration mediators; on the other hand, adhesion molecules may also be a costimulatory signal for T-cell activation by antigen-presenting cells (Wang et al., 2018). Investigations of expressions of adhesion molecules in inflammatory renal diseases indicated up-regulated expression of adhesion molecules in biopsies in cases of chronic histological damage, as well as structural tubular damage and interstitial fibrosis (Tang et al., 2021). In summary, Icam1 is closely correlated with the occurrence and development of renal fibrosis and is a promising target for CKD.

As a human major histocompatibility complex (MHC) also denoted as human leucocyte antigen (HLA), H2-Eb1 (orthologous gene of human HLA-DRB1) is a functional MHC-II gene involved in encoding of the HLA-DR structure chain in the antigen-binding slot. Like CD74, H2-Eb1 has been proved to play a key role in fibrosis, while it is rarely discussed in CKD-related studies. In this study, the up-regulated expression of H2-Eb1 in renal fibrosis was confirmed and it is essentially a promising target gene for the diagnosis and treatment of CKD.

Sequencing techniques of next-generation are powerful methods for detection of novel genes and establishment of transcriptional networks. Herein, transcriptome analysis was applied to UUO mouse models of renal fibrosis in combination with bioinformatics analysis. This study provides more accurate and effective targets for the diagnosis and treatment of CKD.

## Conclusion

Five hub genes were identified and these genes can serve as diagnostic biomarkers in CKD. This study provides accurate and effective targets for the diagnosis and treatment of CKD. Future studies are required to clarify their biological functions in CKD *in vivo*.

## Data Availability

The data presented in this study are deposited in the GEO repository, accession numbers GSE217650 and GSE217654.
