# Supplementary material for: Novel targets in renal fibrosis based on bioinformatic analysis
Source: Front Genet. 2022 Nov 29;13:1046854. doi: 10.3389/fgene.2022.1046854 (PMC9745177; doi:10.3389/fgene.2022.1046854)

# Supplementary material

Supplementary Table 1. The top 30 DEGs in dataset 1.

| id | logFC | AveExpr | t | P.Value | adj.P.Val | B |
| --- | --- | --- | --- | --- | --- | --- |
| Havcr1 | 7.036166 | 5.407656 | 13.67354 | 2.68E-07 | 9.74E-06 | 7.515354 |
| Sprr2f | 6.278622 | 3.421932 | 13.96864 | 2.23E-07 | 8.80E-06 | 7.706801 |
| Lcn2 | 6.222572 | 7.024231 | 14.43812 | 1.68E-07 | 7.54E-06 | 8.00302 |
| C3 | 5.977505 | 5.785697 | 23.91648 | 2.05E-09 | 1.19E-06 | 12.40974 |
| Aoc1 | 5.937882 | 5.11733 | 21.23703 | 5.83E-09 | 1.56E-06 | 11.40336 |
| Spp1 | 5.460082 | 12.26955 | 20.88242 | 6.76E-09 | 1.61E-06 | 11.25866 |
| Timp1 | 5.213624 | 3.709233 | 12.24049 | 6.89E-07 | 1.65E-05 | 6.522515 |
| Mmp7 | 5.207472 | 2.77171 | 9.010427 | 8.83E-06 | 8.59E-05 | 3.805277 |
| Vcam1 | 4.963508 | 4.829249 | 16.58283 | 5.07E-08 | 4.06E-06 | 9.239012 |
| Ccl2 | 4.912092 | 3.703888 | 18.09292 | 2.37E-08 | 2.67E-06 | 10.00974 |
| Pvalb | -4.90246 | 2.495496 | -19.8594 | 1.05E-08 | 1.90E-06 | 10.82439 |
| Krt20 | 4.893548 | 3.226469 | 11.68709 | 1.02E-06 | 2.04E-05 | 6.108271 |
| Cxcl2 | 4.851238 | 2.736522 | 16.97755 | 4.13E-08 | 3.60E-06 | 9.447722 |
| Serpina10 | 4.698179 | 3.765932 | 18.90639 | 1.62E-08 | 2.27E-06 | 10.39568 |
| Col3a1 | 4.671603 | 5.517032 | 17.02075 | 4.04E-08 | 3.57E-06 | 9.470244 |
| Cxcl1 | 4.593903 | 4.553021 | 8.151415 | 1.98E-05 | 0.000154 | 2.938574 |
| Socs3 | 4.5825 | 3.714122 | 22.83634 | 3.08E-09 | 1.40E-06 | 12.02157 |
| Ctss | 4.513903 | 5.556879 | 26.53221 | 8.20E-10 | 9.44E-07 | 13.2641 |
| Sprr1a | 4.497502 | 5.756925 | 5.908082 | 0.000233 | 0.001022 | 0.290199 |
| Hspb1 | 4.45187 | 6.6022 | 15.15059 | 1.11E-07 | 5.85E-06 | 8.434004 |
| Thy1 | 4.445659 | 3.989141 | 12.22465 | 6.97E-07 | 1.66E-05 | 6.510909 |
| Col1a1 | 4.443474 | 5.43218 | 20.45846 | 8.10E-09 | 1.76E-06 | 11.08179 |
| Cd44 | 4.426173 | 3.190815 | 16.43874 | 5.47E-08 | 4.17E-06 | 9.161475 |
| Mgp | 4.374372 | 8.844383 | 15.95874 | 7.07E-08 | 4.68E-06 | 8.897765 |
| Slc22a7 | -4.36241 | 5.207881 | -7.48295 | 3.89E-05 | 0.00025 | 2.211788 |
| Aldh1a2 | 4.346369 | 3.375514 | 21.96233 | 4.34E-09 | 1.47E-06 | 11.69049 |
| Tmsb10 | 4.320038 | 9.451341 | 16.88757 | 4.33E-08 | 3.66E-06 | 9.400618 |
| Cp | 4.31906 | 5.83992 | 16.28441 | 5.93E-08 | 4.33E-06 | 9.0776 |
| Slc7a13 | -4.30152 | 5.934049 | -6.93275 | 7.03E-05 | 0.000394 | 1.575659 |
| Cyp2d12 | -4.29393 | 3.349058 | -12.3907 | 6.21E-07 | 1.55E-05 | 6.63181 |

Supplementary Table 2. The top 30 DEGs in dataset 2.

| id | logFC | AveExpr | t | P.Value | adj.P.Val | B |
| --- | --- | --- | --- | --- | --- | --- |
| Havcr1 | 7.315176 | 5.440486 | 25.56014 | 4.18E-08 | 1.68E-05 | 9.643882 |
| C3 | 6.398427 | 5.635747 | 36.43291 | 3.66E-09 | 7.01E-06 | 11.78476 |
| Lcn2 | 6.096584 | 6.354306 | 19.62403 | 2.54E-07 | 3.02E-05 | 7.868977 |
| Spp1 | 5.70289 | 11.90207 | 13.9867 | 2.51E-06 | 8.55E-05 | 5.472358 |
| Aoc1 | 5.661505 | 5.016662 | 77.73448 | 1.96E-11 | 3.76E-07 | 14.926 |
| Timp1 | 5.488246 | 3.589401 | 26.4603 | 3.30E-08 | 1.67E-05 | 9.866826 |
| Sprr2f | 5.427985 | 2.895976 | 12.31663 | 5.87E-06 | 0.000134 | 4.556774 |
| Vcam1 | 5.135208 | 4.388465 | 55.17174 | 2.09E-10 | 1.34E-06 | 13.77668 |
| Ubd | 5.030323 | 3.850572 | 18.15448 | 4.32E-07 | 3.79E-05 | 7.326949 |
| Krt20 | 5.01299 | 2.91214 | 20.09898 | 2.16E-07 | 2.80E-05 | 8.034039 |
| Ccl2 | 5.01022 | 3.490267 | 19.45724 | 2.70E-07 | 3.07E-05 | 7.809888 |
| Serpina10 | 4.995264 | 3.883478 | 23.80147 | 6.81E-08 | 2.01E-05 | 9.17698 |
| Hspb1 | 4.843531 | 6.56034 | 25.61275 | 4.12E-08 | 1.68E-05 | 9.657197 |
| Cxcl2 | 4.842477 | 2.635767 | 24.69742 | 5.29E-08 | 1.78E-05 | 9.420247 |
| Socs3 | 4.783958 | 3.59166 | 20.17117 | 2.11E-07 | 2.80E-05 | 8.058722 |
| Egf | -4.72878 | 5.606596 | -17.5348 | 5.46E-07 | 4.33E-05 | 7.083 |
| Sprr1a | 4.678521 | 5.992332 | 8.247817 | 8.01E-05 | 0.000648 | 1.690178 |
| Cp | 4.574896 | 5.628488 | 35.12141 | 4.71E-09 | 7.52E-06 | 11.57971 |
| Tmsb10 | 4.498457 | 9.1474 | 16.09831 | 9.75E-07 | 5.34E-05 | 6.478047 |
| Pvalb | -4.49299 | 2.395893 | -28.8179 | 1.84E-08 | 1.46E-05 | 10.40507 |
| Nccrp1 | -4.48358 | 4.175631 | -14.0846 | 2.40E-06 | 8.37E-05 | 5.522459 |
| Il1f6 | 4.402996 | 2.251304 | 8.357571 | 7.37E-05 | 0.000613 | 1.783055 |
| Cd44 | 4.397184 | 2.76873 | 25.54231 | 4.20E-08 | 1.68E-05 | 9.63936 |
| Cyp2d12 | -4.25216 | 4.231492 | -17.0915 | 6.50E-07 | 4.49E-05 | 6.902393 |
| Ctss | 4.169871 | 5.137799 | 13.89352 | 2.63E-06 | 8.80E-05 | 5.424324 |
| Dnase1 | -4.11149 | 7.685426 | -18.204 | 4.24E-07 | 3.79E-05 | 7.346015 |
| Lyz2 | 4.098569 | 5.955657 | 11.31043 | 1.03E-05 | 0.000185 | 3.942512 |
| Cd14 | 4.018493 | 4.16673 | 27.17979 | 2.74E-08 | 1.67E-05 | 10.03783 |
| Ly86 | 4.016447 | 4.145248 | 10.47762 | 1.71E-05 | 0.000244 | 3.39232 |
| C1qa | 4.000784 | 6.232833 | 17.51366 | 5.51E-07 | 4.34E-05 | 7.07451 |

The parameter settings in STRING are as follows picture:


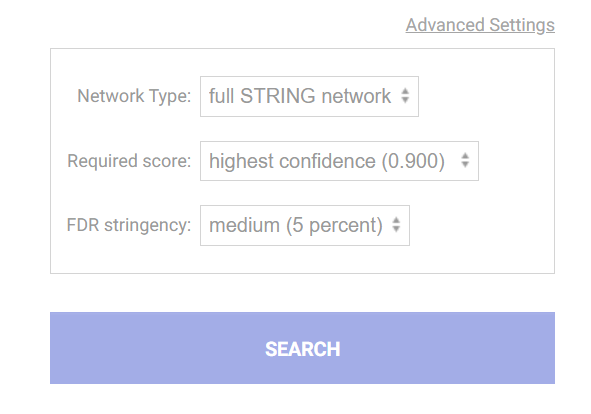


PPI enrichment P value is less than 1.0e-16, as shown in the below:


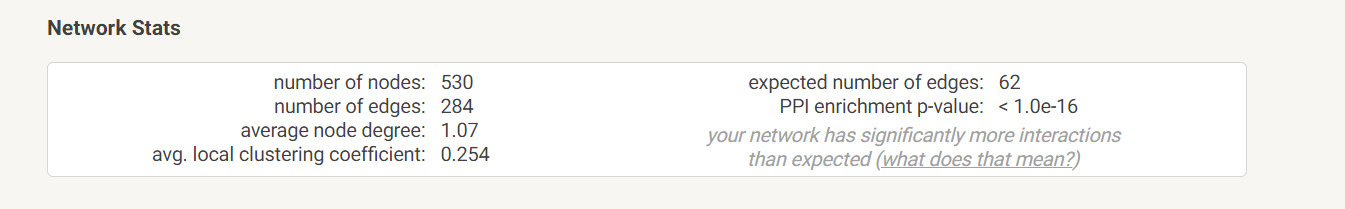


The certification is shown in below


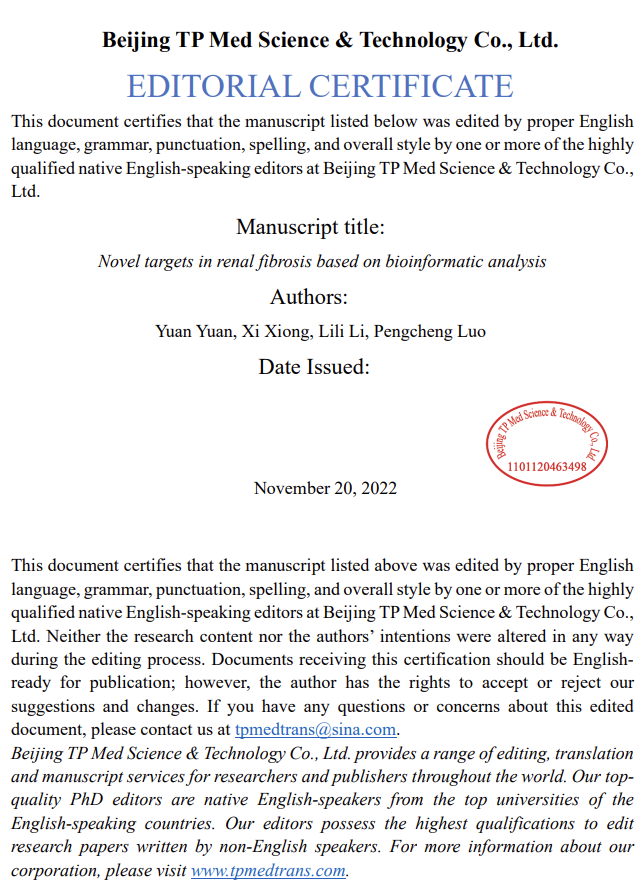

Supplement: Supplementary file 1 [file Table1.DOCX]
